# Inquests after Operations

**Published:** 1910-10-08

**Authors:** 


### INQUESTS AFTER OPERATIONS.

DIFFERENCES of opinion between medical men and coroners as to the certification of fatalities occurring in the course of some mortal disease, but accelerated by some measure designed for the patient's relief, have before now been the subject of comment in these columns. For a long time there was no universal practice even to notify to a coroner a death actually occurring during an operation; indeed, it is still in doubt whether any penalty could attach for failure to do so. But as a matter of public convenience and interest it is now, quite correctly, the authorised and recognised procedure to inform the coroner of all such occurrences, some of which are due to the influence of the anæsthetic; and most coroners make it a rule to hold an inquest. With this custom the medical profession cannot reasonably quarrel, unnecessary as many of these inquests are; for it is most important that the public should not believe, or have any grounds for believing, that anæsthetic or other operation-room fatalities go commonly unreported by connivance of the medical profession.

But a further development of the same principle which has recently made its appearance at Preston does really require an emphatic protest. On September 26 the coroner in that town held an inquest upon the body of a man who died in the Royal Infirmary three hours after an operation. From the medical evidence it seems that the patient was suffering from some grave internal malady demanding as his sole chance of recovery what is known as an emergency operation. According to the surgeons he had but a few hours left to live otherwise, and the chance of saving his life by prompt operation was, though not large, amply sufficient to justify, indeed to demand it. The operation, however, undoubtedly accelerated death by a few hours. The death was not communicated to the coroner, who heard of it accidentally a few days later and at once ordered an inquest. During the proceedings he gave it as his opinion that it is compulsory for him to do this; the doctors combated this view, and had put their theory into practice by giving a certificate. It was pointed out to the coroner that many operations of this sort are performed as forlorn hopes; and we all know that quite a large percentage of such patients are thus saved who would otherwise certainly die. To conduct a public inquiry into every such case when the disease masters the surgeon is not only against the common custom, but against public policy as well. If death three hours after operation is to necessitate an inquest, then logically death three days after—or three weeks after—from, say, embolism or bronchitis must do the same; and the number of coroners' inquests will increase twenty-fold. The contention is, in our deliberate judgment, quite untenable; it renders medical and especially surgical practice so harassing that no man in his senses will accept the responsibilities. To pooh-pooh, as this coroner does, that aspect of the situation is as unreasonable as his action is unfair. The coroners have to rely mainly on the medical profession for the notification of anæsthetic fatalities, poisoning cases, and many other forms of violent and unnatural death; and they will do well not to set themselves too violently against informed medical public opinion. To suggest that death following operation for such a disease as general peritonitis is unnatural is a ridiculous attitude, and one that must be promptly repudiated on behalf of the profession.

